# Comparative Evaluation of Horizontal Lip Position With Ricketts Esthetic Plane in Preschool and School-Going Children of Bankura, West Bengal: A Craniofacial Anthropometric Study

**DOI:** 10.7759/cureus.55015

**Published:** 2024-02-27

**Authors:** Annapurna Ahuja, Vipin Ahuja, Jaya Verma, Arunima Arunima, Nilima R Thosar

**Affiliations:** 1 Periodontics and Implant Dentistry, Hazaribag College of Dental Sciences and Hospital, Hazaribag, IND; 2 Pediatric and Preventive Dentistry, Government Dental College and Hospital, Jamnagar, IND; 3 Pediatric and Preventive Dentistry, Hazaribag College of Dental Sciences and Hospital, Hazaribag, IND; 4 Pediatric and Preventive Dentistry, Sharard Pawar Dental College and Hospital, Datta Meghe Institute of Higher Education and Research (Deemed to be University), Wardha, IND

**Keywords:** mixed dentition, primary teeth, esthetic line, ricketts e-line, lateral profile, anthropometry

## Abstract

Introduction

The horizontal lip position and esthetic plane are two important parameters to define facial beauty, and these factors are always given importance in children undergoing fixed orthodontic therapy. The purpose of this study was to evaluate horizontal lip position in primary and mixed dentition children with class I occlusion and to analyze its association with gender among preschool and schoolchildren of villages in the Bankura district in West Bengal.

Materials and methods

Researchers screened 437 children for the study and selected those who met the inclusion criteria. A total of 407 children were segregated: 201 children aged three to five years with the flush terminal plane and mesial step in primary teeth and 206 children aged seven to eleven years with class I occlusion in mixed dentition were selected from schools in villages in Bankura district, West Bengal, India. The subjects were instructed to hold the head in the natural head position by looking straight, and points were marked on the nose and chin tip, respectively. A metallic ruler was placed from nose to chin, representing Rickett's esthetic line. The horizontal lip distance to the esthetic plane of both upper and lower lips was measured as a linear distance from the most anterior part of the lip to the metallic ruler. The data were recorded, compared with gender, and statistically analyzed using the Chi-square test using the Statistical Package of Social Sciences software (SPSS version 19.0, 2015, IBM Corp., Armonk, NY).

Results

The most prevalent horizontal lip distance for both upper and lower lips to the esthetic line for primary and mixed dentition in the Bankura region of West Bengal was category I, where the lip is seen beyond the E plane, followed by category II, where lips are at a horizontal distance in the range of 0-1.5 mm from the esthetic line. A significant correlation of lip position with Rickets aesthetic plane was also illustrious with gender in primary dentition, but a non-significant association with gender was noted in mixed dentition.

Conclusion

Children with class I occlusion from the Bankura district of West Bengal showed a higher percentage of lip position beyond the esthetic plane in both primary and mixed dentition, which is not in line with the inference of Ricketts's studies on the Caucasian population. There is a definite association between gender and horizontal lip distance in Ricketts esthetic plane. Protrusive upper lips were seen more in males, and retrusive lips were seen more in females.

Clinical significance

The horizontal lip position with reference to Ricketts esthetic plane has been documented in the literature for adults and teens undergoing fixed orthodontic treatment. However, there is no study done to define these measurements in preschool and school-going children, which can assist in determining future esthetic profiles and in preparing a protocol for early age interceptive orthodontics along with aesthetic rehabilitation of the anterior area of the mouth.

## Introduction

Human face anthropometry is a prospective science that is growing every year with increasing demands for aesthetics and beauty. With the advent of modern technology and advanced anthropometric techniques like digital anthropometry and 3D face scanning, the dynamic field has entered numerous fields of medicine and dentistry. Some of the noteworthy applications, apart from research, include identification prospects in forensic dentistry, assessment prospects in the growth and development of children, and treatment prospects in cleft lip and palate and post-trauma facial reconstructions. Children are extremely conscious of their looks, and this makes this branch imperative for the pediatric dentist. As the paradigm shifts from late-age orthodontics to early age pediatric orthodontics, the importance of soft tissue analysis in children is gaining more value. In addendum to this, the balance between facial harmony and aesthetics becomes a key factor in planning orthodontic treatment for growing children [1].

Robert Ricketts, in 1950, put the spotlight on the importance of equating the position of the lips, nose, chin, and other soft tissues of the face from an aesthetic profile standpoint. He highlighted the importance of the “esthetic plane,” or “E” plane, which, according to him, was not given much importance during orthodontic alignment and ultimately led to a worsening of facial appearance. The "esthetic" or "E" plane is a line drawn from the tip of the nose to the tip of the chin. Ricketts studied the Caucasian race and inferred that in an average face with a pleasing facial profile, the upper lip should be 4 mm and the lower lip should be 2 mm behind the "E" plane. The linear relationship of lips with the esthetic plane is important and has distinguished clinical applications in pediatric dentistry, like treatment planning of dental crowding in fixed pediatric orthodontics and selection of tooth size and shade in pediatric restorative dentistry. If lips are beyond the esthetic plane, extraction with anterior retraction could be considered in the treatment protocol, and if lips are behind the esthetic plane, a non-extraction method with arch expansion could be considered. If lips are beyond or closer to the esthetic line than normal values, the selected tooth size should be greater than average, as lips and teeth will dominate and govern the smile. On the contrary, if lips are far behind the esthetic line, the selected tooth size should be less than average with a brighter shade as the nose and chin gain more importance in smile fundamentals [2,3].

The prime need for conducting this study was to quantify the horizontal distance between lips and Ricketts esthetic plane and to evaluate the outcomes in preschool and school-going children in Bankura. Second, it was intended to correlate the outcomes with baseline data established by Dr. Ricketts on the esthetic plane. Third, the research was conceived to correlate study parameters with sexual dimorphism in these children. Literature is in overabundance to prove that many authors have used different lines and planes to determine the horizontal position of lips in an antero-posterior direction to esthetic planes to accentuate orthodontic treatment planning. However, the base of this research was the Caucasian population, and the outcome was generalized globally. There are few studies that document that these results vary with varied races and ethnicities [3]. The Bankura district was chosen for this study because a specific tribal community speaking the Santhal language resides in this region. The anthropometric parameters chosen in this study vary with different communities, races, and ethnicities. Therefore, we chose a specific community as a sample, representing the Santhal population. Moreover, there is a weak source of information on this aspect in preschool and school-going children of the Indian race, and as far as our knowledge goes, this is the first study where lip distance to the esthetic "E" plane was recorded for both primary and mixed dentition comprehensively in children from Bankura district of West Bengal.

## Materials and methods

Study area

The study area was the private schools under the care of Shaimayitah Math in Ranabahai village in the Bankura district of West Bengal. In order to avoid information bias, a well-trained, single examiner from the Department of Pediatric and Preventive Dentistry, Hazaribag College of Dental Sciences and Hospital, Hazaribag, who did the pilot study with the same parameters, was appointed for the study in these schools.

Sample size calculation

The sample size calculation was done using the software G*Power 3.1.9.4. Based on the reference article, the effect size was kept at 0.41, the alpha error was 5%, and the power of the study was 80%. For the chi-square test, the total sample size required for the study was 88 for both male and female groups. So, to be on the safe side, we have taken this sample size.

Sample selection

The foundation of this craniofacial anthropometric clinical study was the examination of the children in pre-school and school age with primary dentition and mixed dentition, respectively. The sample selection was done using the stratified sampling method, where participants were screened for occlusion criteria and those with class I molar relationships were segregated and screened for further inclusion criteria to finalize the study sample.

Ethical approval

The Institutional Ethical Committee of Hazaribag College of Dental Sciences and Hospital, Hazaribag, has given ethical approval for this study (HCDSH/ADM/BNF/2020/215).

Inclusion and exclusion criteria

The study participants of pure Bengali ethnic origin were selected, and ancestral history was recorded to confirm their ethnicity. For preschool children, participants with a full set of primary dentition and without any partially/completely erupted permanent teeth, with no previous orthodontic treatment or craniofacial trauma or rehabilitation history, were selected for the study. For school-going children, participants with mixed sets of primary dentition and permanent dentition and with the obligatory presence of the first permanent molar were included. Children with lip asymmetries, a history of lip surgery, reconstruction, or on-going orthodontic treatment, and craniofacial syndromes were excluded from the study.

Data collection

**Figure 1:**
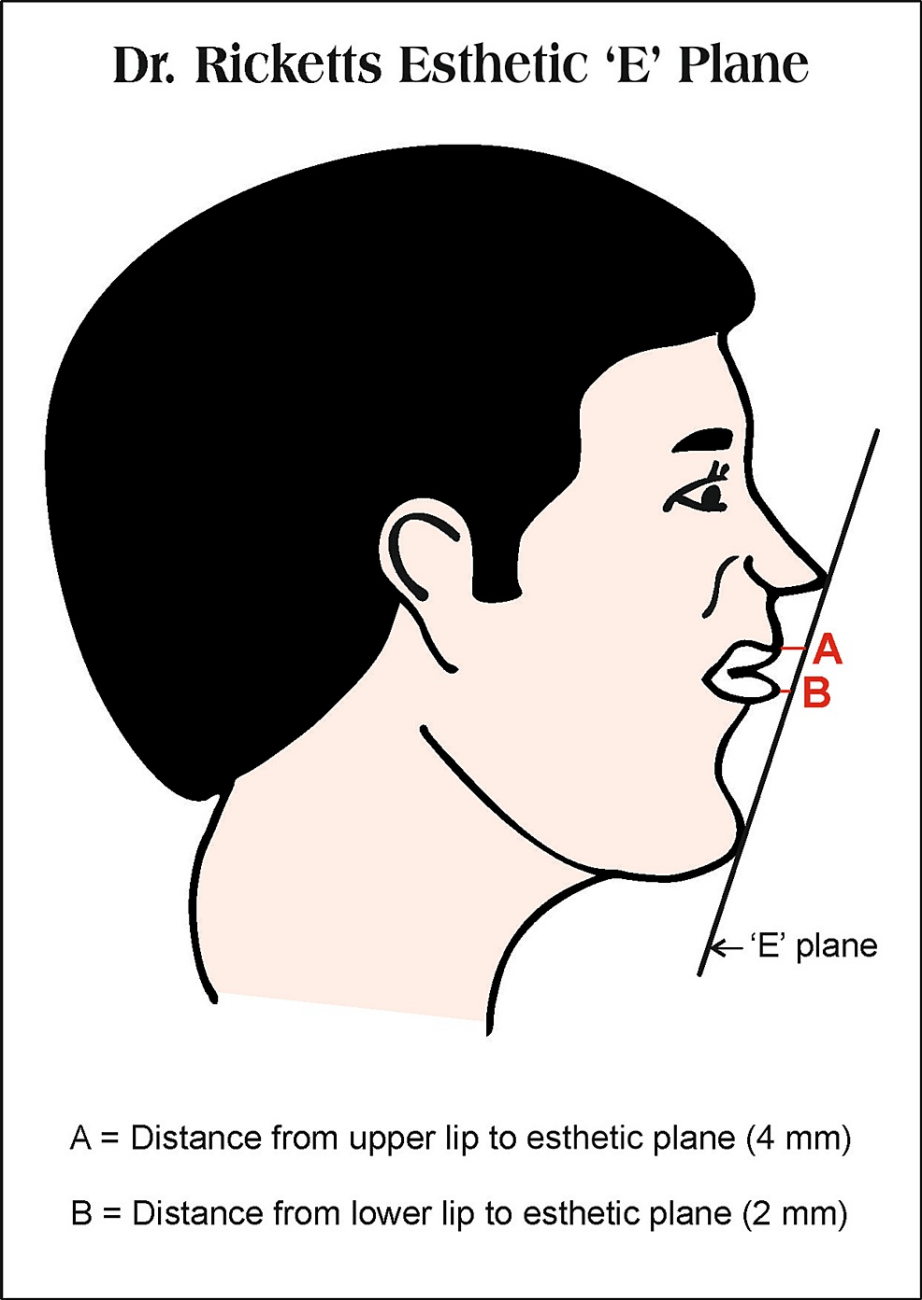
Ricketts esthetic 'E' plane and horizontal distance relationship with upper lips (A = 4 mm) and lower lips (B = 2 mm) Image credit: Dr. Vipin Ahuja

A well-trained, single examiner from the Department of Pediatric and Preventive Dentistry examined children in their corresponding schools with type IV examinations under good daylight. Prior to the study, written consent was obtained from parents, and in addendum to this, verbal assent was also obtained from children selected for the study. The study protocol was properly discussed with the school principal, and the research was initiated after his approval. Children were made to rinse their mouths with water prior to the examination. Then, they were instructed to sit in a relaxed position on the chair and tutored to bite in centric occlusion, and the occlusion was recorded. The subjects were instructed to hold the head in the natural head position by looking straight, and points were marked with a blue ballpoint pen on the nose and chin tip, respectively. A metallic ruler was placed manually from the most anterior point of the nose to the most anterior point of the chin, representing Ricketts esthetic plane (Figure 1). The diagrammatic representation of Ricketts "E" plane and the distance measurement of lips to the "E" plane are shown.

**Figure 2:**
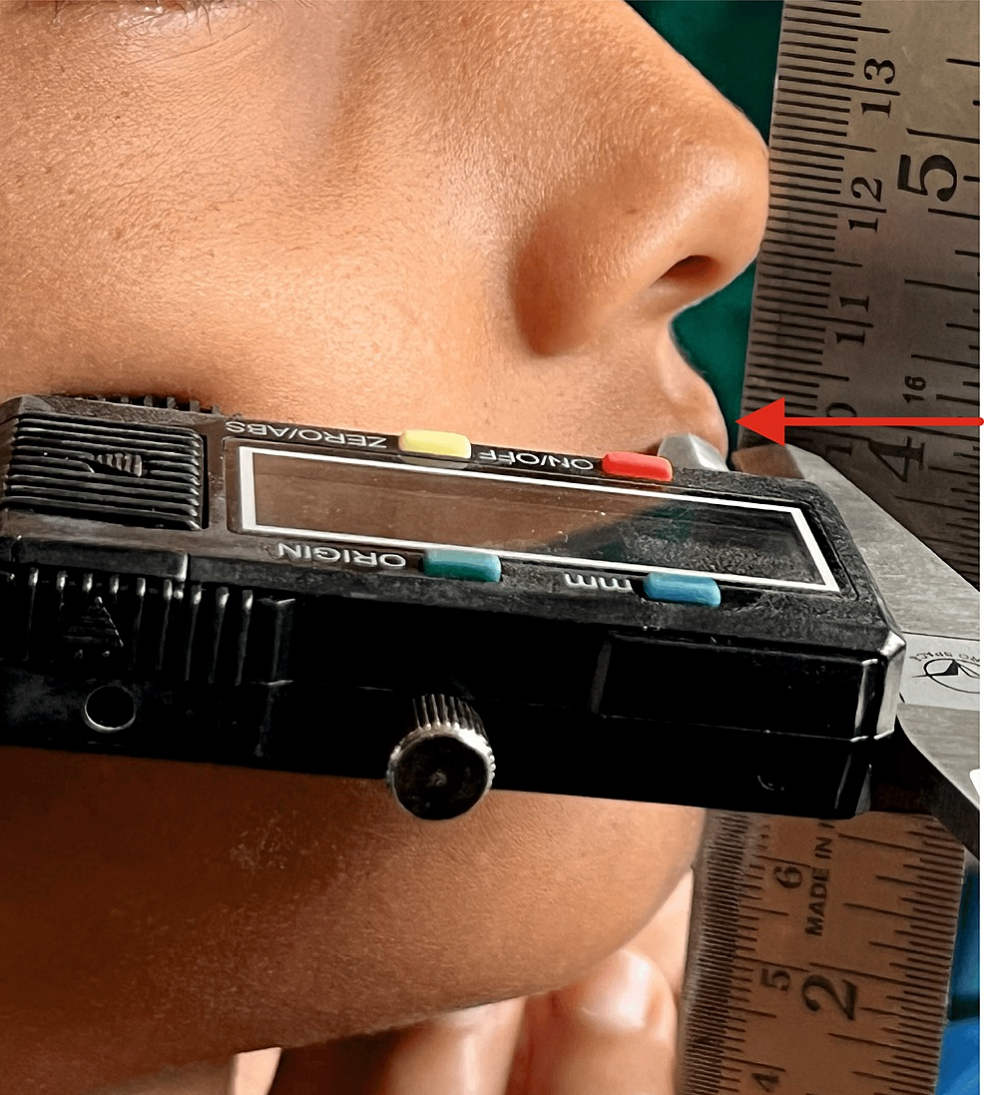
Clinical recording of horizontal lip position of upper lip to Ricketts esthetic plane

The horizontal lip distance to the esthetic plane of both upper and lower lips was measured as a linear distance from the most anterior point of the lips to the first metallic ruler (Figure 2). The clinical recording of horizontal lip distance to Ricketts esthetic plane is shown with a digital vernier caliper for the upper lips.

**Figure 3:**
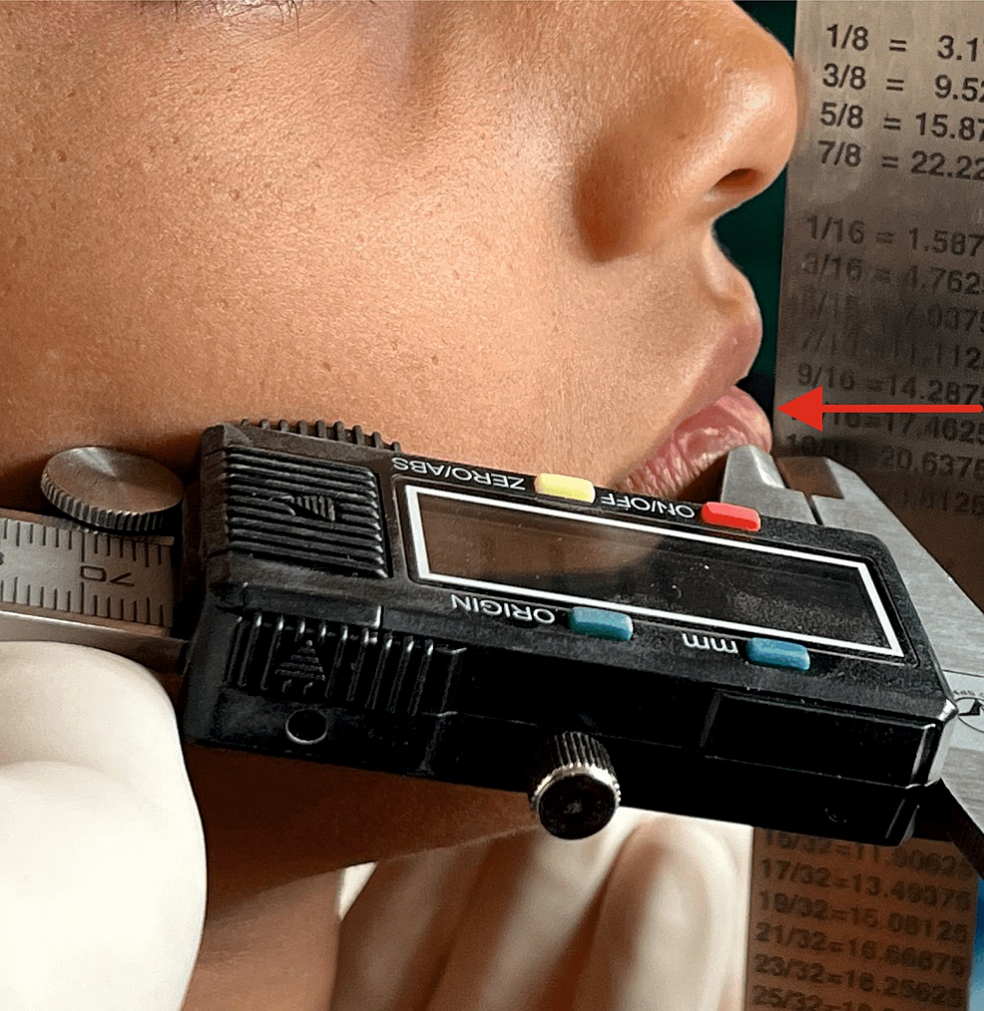
Clinical recording of horizontal lip position of lower lip to Ricketts esthetic plane

The clinical recording of horizontal lip distance to Ricketts esthetic plane is shown with a digital vernier caliper for lower lips (Figure 3).

**Figure 4:**
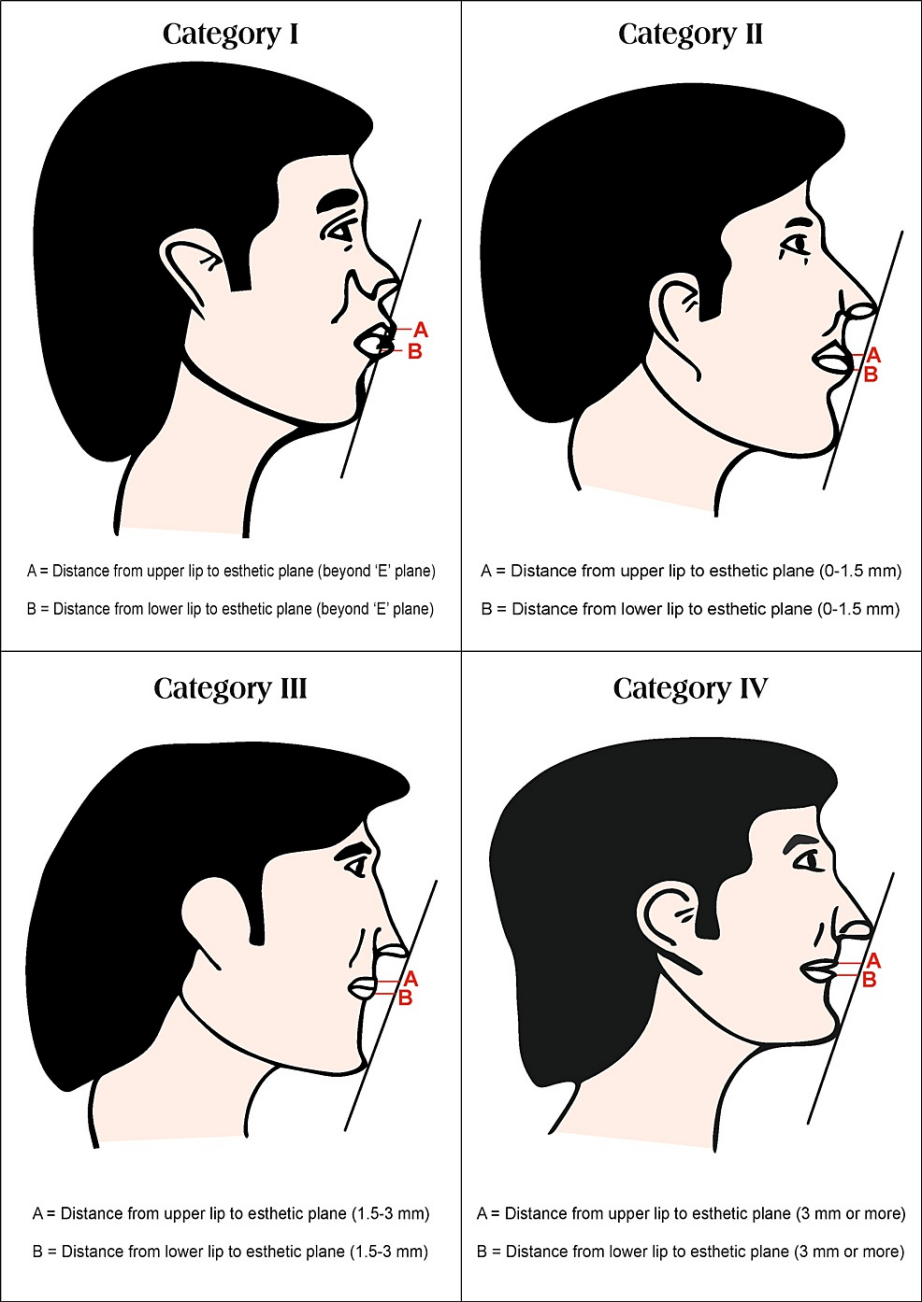
Category wise representation of Ricketts "E" plane and horizontal distance relationship with upper and lower lips Image credit: Dr. Vipin Ahuja

The measurements were confirmed with a digital vernier caliper. The horizontal lip distance measurement was recorded with three readings, and the mode of these readings was taken as the final reading to enable cross-checking of measurements. Based on horizontal lip distance to the "E" plane, the subjects were categorized into four categories as follows: (i) category I - lips are beyond Ricketts esthetic plane; (ii) category II - lips are 0 to 1.5 mm behind Ricketts esthetic plane; (iii) category III - lips are 1.5 mm to 3 mm behind Ricketts esthetic plane; (iv) category IV - lips are 3 mm or more behind Ricketts esthetic plane. The four categories of horizontal distance relationships to Ricketts "E" plane are diagrammatically shown (Figure 4).

Statistical analysis

The data obtained was subjected to statistical analysis with the consultation of a statistician. The data obtained were compiled systematically, and the master chart was sent to the statistician. The data collected were entered, and statistical analysis was done using Chi-square tests. The level of significance was set at 0.05. Statistical analysis was done using the Statistical Package of Social Sciences (SPSS Version 19.0; Chicago, Inc., USA, 2015).

## Results

A total of 407 children were segregated for the study. Out of that, 203 males and 204 females were examined for horizontal lip position on the Ricketts E plane in different categories of different types for primary and mixed dentition occlusion. The correlation of this parameter was also analyzed with gender.

The sex-wise distribution of the horizontal lip position from the upper lip to the Ricketts E plane in primary dentition is shown (Table 1). The most prevalent horizontal lip distance to the esthetic plane is in category I (77.6%), where the lip is seen beyond the E plane, followed by category II (12.4%), category IV (7%), and category III (3%), respectively, and the results were statistically significant. A significant correlation was also noted with gender. The prevalence of category I, where lip is seen beyond the E plane, is higher in males (88.7%) when compared to females (65.3%), and the order of occurrence of lip categories was the same in both sexes.

**Table 1:** Evaluation of upper lip to Ricketts esthetic plane in preschool children with primary dentition X² = 16.58, P = 0.001, S

Categories	Female		Male		Total	
	No.	%	No.	%	No.	%
Category I: Beyond the esthetic plane	62	65.3	94	88.7	156	77.6
Category II: 0–1.5 mm behind the esthetic plane	19	20.0	6	5.7	25	12.4
Category III: 1.5–3.0 mm behind the esthetic plane	5	5.3	1	0.9	6	3.0
Category IV: >3.0 mm behind the esthetic plane	9	9.5	5	4.7	14	7.0
Total	95	100.1	106	100.0	201	100.0

The sex-wise distribution of the horizontal lip position from the lower lip to the Ricketts E plane in primary dentition is shown. The most prevalent horizontal lower lip distance to the esthetic line is in category I (84.1%), where the lower lip is seen beyond the E plane, followed by category II (10.4%), category III (5%), and category IV (0.5%), respectively, and the results were statistically significant. A significant correlation was also noted with gender. The prevalence of category I, where the lip is seen beyond the E plane, is higher in males (88.7%) when compared to females (79%), and the order of occurrence of lip categories was the same in both sexes (Table 2).

**Table 2:** Evaluation of lower lip to Ricketts esthetic plane in preschool children with primary dentition X² = 12.62, P = 0.006, S

Categories	Female		Male		Total	
	No.	%	No.	%	No.	%
Category I: beyond the esthetic plane	75	79.0	94	88.7	169	84.1
Category II: 0–1.5 mm behind the esthetic plane	10	10.5	11	10.4	21	10.4
Category III: 1.5–3.0 mm behind the esthetic plane	10	10.5	0	0.0	10	5.0
Category IV: >3.0 mm behind the esthetic plane	0	0.0	1	0.9	1	0.5
Total	95	100.0	106	100.0	201	100.0

The sex-wise distribution of the horizontal lip position from the upper lip to the Ricketts E plane in mixed dentition is shown. The most prevalent horizontal lip distance to the esthetic line is category I (66.1%), where the lip is seen beyond the E plane, followed by category II (15.5%), category III (15%), and category IV (3.4%), respectively. A non-significant correlation was also noted with gender. The prevalence of category I, where the lip is seen beyond the E plane, is higher in males (71.1%) when compared to females (61.4%), and the order of occurrence of lip categories was the same in both sexes (Table 3).

**Table 3:** Evaluation of upper lip to Ricketts esthetic plane in school children with mixed dentition X² = 2.90, P = 0.41, NS

Categories	Female		Male		Total	
	No.	%	No.	%	No.	%
Category I: beyond the esthetic plane	67	61.4	69	71.1	136	66.1
Category II: 0–1.5 mm behind the esthetic plane	21	19.3	11	11.3	32	15.5
Category III: 1.5–3.0 mm behind the esthetic plane	17	15.6	14	14.4	31	15.0
Category IV: >3.0 mm behind the esthetic plane	4	3.7	3	3.2	7	3.4
Total	109	100.0	97	100.0	206	100.0

The sex-wise distribution of the horizontal lip position from the lower lip to the Ricketts E plane in mixed dentition is shown. The most prevalent horizontal lip distance to the esthetic line is in category I (86.8%), where the lip is seen beyond the E plane, followed by category II (8.3%) and category III (4.9%), respectively. A non-significant correlation was also noted with gender. The prevalence of category I, where the lip is seen beyond the E plane, is higher in males (87.7%) when compared to females (86.2%), and the order of occurrence of lip categories was the same in both sexes (Table 4).

**Table 4:** Evaluation of lower lip to Ricketts esthetic plane in preschool children with mixed dentition X² = 0.21, P = 0.90, NS

Categories	Female		Male		Total	
	No.	%	No.	%	No.	%
Category I: beyond the esthetic plane	94	86.2	85	87.7	179	86.8
Category II: 0–1.5 mm behind the esthetic plane	9	8.3	8	8.2	17	8.3
Category III: 1.5–3.0 mm behind the esthetic plane	6	5.5	4	4.1	10	4.9
Category IV: >3.0 mm behind the esthetic plane	0	0	0	0	0	0
Total	109	100.0	97	100.0	206	100.0

## Discussion

Lips are the soft appendages that form an invincible module of facial esthetics and functionality in human beings. "Labium superius oris" and "Labium inferius oris" are the terms used to denote the upper and lower lips, respectively. The dimensions, position, and fullness of the lip in relation to facial width determine a vital segment of defining beauty and looks in growing teens and adults. However, this varies with different ages, races, and ethnicities. Many authors in the past have defined many linear measurements and proportions to define ideal lip length and position. One such line is the "Ricketts E plane," given by Dr. Robert Ricketts, and is a line drawn from the tip of the nose to the tip of the chin on the lateral profile of the face [4-6]. In our study, the Ricketts E plane was chosen as the appropriate reference line, as this plane was typically used in the past by many researchers studying facial aesthetics with orthodontic diagnosis and treatment planning. There is a plethora of literature supporting the Ricketts E plane as the most suitable linear measurement and its relation to lips as a significant factor defining attractiveness in both sexes. In addition to this, the use of the E plane is practically applicable, easy, and convenient to use, and it is of pertinent value in silhouette studies as anatomical landmarks are placed in the anterior region. It can also be used to set an appropriate point for comparing varied races and ethnic communities, as nasal prominence is a specific characteristic of the face and varies with varied populations [7-9]. In our methodology, direct measurement anthropometry was used instead of photographic anthropometric analysis, which was in line with a study by Franke-Gromberg et al., where it was inferred that direct measurement is more reliable and that in photographic kephalometry, images are 7.6% shorter than normal. Weinberg et al. did a study to compare precision in direct anthropometry with digital 3D photogrammetry systems, and it was concluded that no significant difference was noted in precision, and direct anthropometric measurements are fairly equal to 3D anthropometry [10,11].

Association of horizontal lip position with Ricketts esthetic plane

Upper Lip

The majority of preschool and school-going children in primary and mixed dentition fall in category I, where the upper lip is seen beyond the E plane, followed by category II, where the lip is behind the E plane by a range of 0-1.5 mm, category IV, where the lip is behind the E plane by more than 3 mm, and category III, where the lip is behind the E plane by a range of 1.5-3 mm.

Lower Lip

The majority of preschool and school-going children in primary and mixed dentition fall in category I, where the lower lip is seen beyond the E plane, followed by category II, where the lip is behind the E plane by a range of 0-1.5 mm, category III, where the lip is behind the E plane by a range of 1.5-3 mm, and followed by category IV, where the lip is behind the E plane by more than 3 mm. The results indicate that West Bengali children of the Bankura region showed upper and lower lips beyond Ricketts esthetic plane. This observation is an unpleasant sign of facial esthetics and may require premolar extraction in fixed orthodontic procedures. Our study findings are in line with a handful of previous studies. Isiekwe et al. concluded in a Nigerian cephalometric study that both upper and lower lips were more protrusive than the normal values mentioned for Ricketts E‑plane and inferred that the Nigerian sample has more protrusive lips than Caucasians. Ghorbanyjavadpour and Rakhshan, in their studies, inferred that protrusive lips, rather than those ideal for Ricketts E plane, seem more desirable and attractive in youth, and therefore, procumbent lips are preferable in the Indian population. Also, Polk et al. reported that preferred profiles were anterior to the E plane in their study. Wilson et al. found that in young black South African women, lips showed increased protrusion beyond the Ricketts E-line [8,12-14]. It has been mentioned in literature that protrusive lips are seen in Chinese and Turks when compared to Yemenis and Americans. Farrow et al. also conducted research where 15 black subjects were randomly selected and lateral photographs were taken, followed by computer alterations for different profiles. It was found that a slightly convex profile with protrusive lips is more attractive than white orthodontic norms. In an elusive study by Peck and Peck, it was highlighted that general public agreement on facial profile regarding attractiveness is more important than orthodontic norms for facial beauty. It was concluded that the general public admires a fuller and more protrusive dentofacial pattern [15,16].

Association of horizontal lip position with sexual dimorphism

Upper Lip

In preschool and school-going children with primary and mixed dentition, a significant correlation was also noted with gender. The highest prevalence of horizontal lip position on the Ricketts plane falls in category I, where the lip is ahead of the Ricketts plane. Males showed a higher prevalence of lips seen beyond the E plane (category I) when compared to females (77.6% in primary dentition, 66.1% in mixed dentition); however, in the rest of the population where lips are seen behind the E plane (categories II, III, and IV), females dominated males. The results indicate that protrusive upper lips are seen more in males, and retrusive lips are seen more in females.

Lower Lip

In preschool with primary dentition, a significant correlation was also noted with gender. The highest prevalence of horizontal lip position on the Ricketts plane falls in category I, where the lip is ahead of the Ricketts plane. Males showed a higher prevalence of lip seen beyond the E plane (category I) when compared to females (84.1%). However, in the rest of the population, where lips are seen behind the E plane (categories II, III, and IV), females have dominated males. In school-going children with mixed dentition, a non-significant association is seen with gender. The results indicate that protrusive lower lips are seen more in males and retrusive lips are seen more in females in preschool and school-going age. The sexual dimorphism regarding lip length, volume, and structure has been mentioned in a plethora of previously documented literature [5,8,16-20]. Mamandras et al. found significant sexual dimorphism and inferred that male lips are larger than female in all measurements [21]. Rakhshan also reported that, in Iranian people, males have more protrusive upper lips and females have retrusive lower lips, which goes in agreement with our study. On the contrary, there are studies that show no significant sexual dimorphism in Americans and Brazilians [22,23]. Otuyemi et al. [7], Yehezkel et al. [24], and Bishara et al. [25] also highlighted that young people like protrusive and fuller lips, whereas mid-age and older groups prefer flatter lips in their profiles [7,24-25]. To summarize, Ricketts's measurements were the outcome of significant research on European Whites, and so the norms may not be the ideal values for different populations like Indians, and variations can exist with regional and cultural diversities. In our study, the majority of children showed both upper and lower lips beyond Ricketts esthetic line, and this finding is supported by the above-mentioned literature. Sexual dimorphism was also evident in our study. Protrusive lips are seen in a higher percentage of male children than in female children, whereas retrusive lips are seen in higher percentages in females. This finding is analogous to ample research conducted in the past.

Clinical applications and future implications

The study has truly eloquent clinical applications as far as pediatric dentistry is concerned. Ricketts esthetic plane and its horizontal distance relationship with both upper and lower lips are imperative to analyze facial beauty and harmony. As per standardized norms, lips in an attractive face should lie behind the esthetic plane, and if lips are beyond the esthetic plane, extraction of premolars is usually required in crowding or malocclusion cases. With this study, we can contemplate that if lips are beyond the esthetic plane in preschool and school-going children, then the child most probably becomes an extraction case if dental malocclusion occurs. Diagnosing it at an early age will give pediatric dentists ample opportunity to treat these crowding cases through arch preservation and myofunctional appliance therapies. The second clinical application is the selection of tooth size for anterior teeth in partial dentures in children. If lips are beyond the esthetic plane, a smaller size than average shall be selected to compensate for large and dominant lips, and vice versa. The future implications of this study suggest that the horizontal lip relationship to the Ricketts plane is a significant spot to be understood before planning esthetic dentistry and pediatric orthodontics in children, but there has been no study undertaken to define these measurements in preschool and school-going children. So, this research on young children can be a useful piece of information in determining future esthetic profiles and in preparing protocols for early age interceptive orthodontics and restorative pediatric dentistry.

Strengths and limitations of the study

The biggest strength of our study is that this is the first-ever research conducted on this topic among young children of preschool and school age. The previous studies have evaluated lip distance to the esthetic plane for adolescents and adults as a diagnostic protocol in orthodontics and esthetic dentistry, but this research has quantified and evaluated linear distances of lips to the esthetic plane in young children with primary and mixed dentition, and this has not been reported before. The second strength is the homogeneity of the sample population chosen for the study, which is the utmost requite for anthropometric research with parameters varying with varied populations. The sample population selected belongs to the Santhal language-speaking tribal community of Bankura district, West Bengal, India. The third asset is the adequate sample size, which was much larger than calculated by the statistician during the pilot study. The fourth forte is the standardization protocol of the study. Ricketts E‑plane was chosen as the suitable reference line as this plane is a standardized plane used by many researchers before and it is used in this study to describe distance from esthetic plane to lips. This plane is also practically applicable, easy, and convenient to use. Direct anthropometric methods are used in the study as these methods are economically cheaper, easily available, and equally efficacious as other anthropometric techniques. However, there are a few limitations to this study. The first and foremost limitation is the comparison with gold standard values on this subject. This study is the leading exploration evaluating horizontal lip positioning in reference to Ricketts E plane in preschool and school-going children. Hence, the literature is too sparse to compare and analyze the outputs with previous data. The second one is the generalization of results, as the facial parameters studied were confined to the children of the West Bengal region, and these parameters vary with different populations, races, and ethnicities; therefore, more studies are advised to be done on this subject across the globe to identify ideal values to define facial aesthetics at an early age. The third limitation is the growth and development of children. As children are in a growing phase of their age, the results at the preschool age may not reflect in the same manner when these children enter adulthood. Therefore, longitudinal studies are advisable for these young children to establish noteworthy outcomes.

## Conclusions

The ideal values of horizontal lip positioning in Ricketts's aesthetic plane were the outcome of research on the Caucasian population, which fits a plethora of groups, regions, races, and populations but, at the same time, does not fit many regions and populations. Therefore, the generalization of Ricketts outcome should not be done in all populations. And a large number of studies on varied races and communities are advised in this field to quantify the horizontal relationship of lips to esthetic lines globally. In this study, children from the Bankura district of West Bengal with class I dental occlusion showed the highest percentage of lip position beyond the esthetic plane in both primary and mixed dentition. Moreover, there was a significant association found with gender with both upper and lower lips.
